# Comparative Analyses of Anatomical Structure, Phytohormone Levels, and Gene Expression Profiles Reveal Potential Dwarfing Mechanisms in Shengyin Bamboo (*Phyllostachys edulis* f. *tubaeformis*)

**DOI:** 10.3390/ijms19061697

**Published:** 2018-06-07

**Authors:** Tao Wang, Lei Liu, Xiaojing Wang, Lixiong Liang, Jinjun Yue, Lubin Li

**Affiliations:** 1 State Key Laboratory of Forest Genetics and Tree Breeding, Key Laboratory of Silviculture of the State Forestry Administration, Research Institute of Forestry, Chinese Academy of Forestry, Beijing 100091, China; wangtao5757@126.com (W.T.); liulei519@caf.ac.cn (L.L.); wangxjwork@163.com (X.W.); llx0613@126.com (L.L.); 2 Research Institute of Subtropical Forestry, Chinese Academy of Forestry, Fuyang 311400, China

**Keywords:** dwarf moso bamboo, transcriptome analysis, dwarfing regulation

## Abstract

Moso bamboo (*Phyllostachys edulis*) is one of the most important bamboo species in China and the third most important plant species for timber production. However, the dwarf variant of moso bamboo, *P. edulis* f. *tubaeformis* (shengyin bamboo), which has shortened internodes, is not well studied. We used anatomical, hormonal, and transcriptomic approaches to study internode shortening and shoot growth in dwarf shengyin and wild moso bamboo. Phenotypic and anatomical observations showed that dwarfing in shengyin bamboo is due to reduced internode length, and the culm fibers in shengyin bamboo are significantly shorter and thicker than in wild moso bamboo. We measured the levels of endogenous hormones in the internodes and found that shengyin bamboo had lower levels of four hormones while two others were higher in wild moso bamboo. Comparative transcriptome analyses revealed a potential regulating mechanism for internode length involving genes for cell wall loosening-related enzymes and the cellulose and lignin biosynthesis pathways. Genes involved in hormone biosynthesis and signal transduction, especially those that showed significant differential expression in the internodes between shengyin and wild moso bamboo, may be important in determining the shortened internode phenotype. A hypothesis involving possible cross-talk between phytohormone signaling cues and cell wall expansion leading to dwarfism in shengyin bamboo is proposed. The results presented here provide a comprehensive exploration of the biological mechanisms that determine internode shortening in moso bamboo.

## 1. Introduction

Bamboo is known to be one of the world’s fastest-growing plants. Under natural conditions, culms of developing moso bamboo (*Phyllostachys edulis* (Carr.) H. de Lehaie.) reach their final height of more than ten meters within a short period of 2–4 months [1]. Moreover, moso bamboo is the most important bamboo species in China and the third most important plant species for timber production [1,2,3].

To better understand the growth characteristics and physical properties of bamboo, the anatomical structure of the culms and sequential elongation of the internodes from the base to the top has been observed [3,4]. Transcriptome sequencing and proteomics have enabled studies of the molecular mechanisms underlying the rapid internode elongation, and also prediction of some internode elongation-associated proteins and genes such as fructose 1,6-bisphospate aldolase (FBP) [5], *CYP714D1*/*EUI1* (elongated uppermost internode), ACO1 (1-aminocyclopropane-1-carboxylic acid oxidase 1), *GLU* (glucoamylase) in many plants of Bambusoideae [3,4,6,7,8]. Nevertheless, very few studies have focused on the dwarf characteristic in bamboo, and to date there have been no reports describing the molecular biological mechanisms controlling dwarfing and internode shortening in bamboo.

Dwarfing is one of the most important traits in plant breeding, and it plays an important role in enhancing lodging resistance in some crops. Molecular studies on stem dwarfing and shortened internodes have mainly been in Arabidopsis [9], rice (*Oryza sativa*) [10,11,12] and several other species [13]. Many dwarfing-related genes from the brassinosteroid (BR), gibberellin (GA), and cellulose and fiber elongation hormone biosynthesis and signal transduction pathways have been cloned and overexpressed [14,15], which could provide a theoretical basis for the application of mutant dwarfing genes to crop breeding. Nevertheless, in bamboo, most studies have been conducted on the rapid growth and high fiber content characters, with little focus on dwarfing.

**Figure 1:**
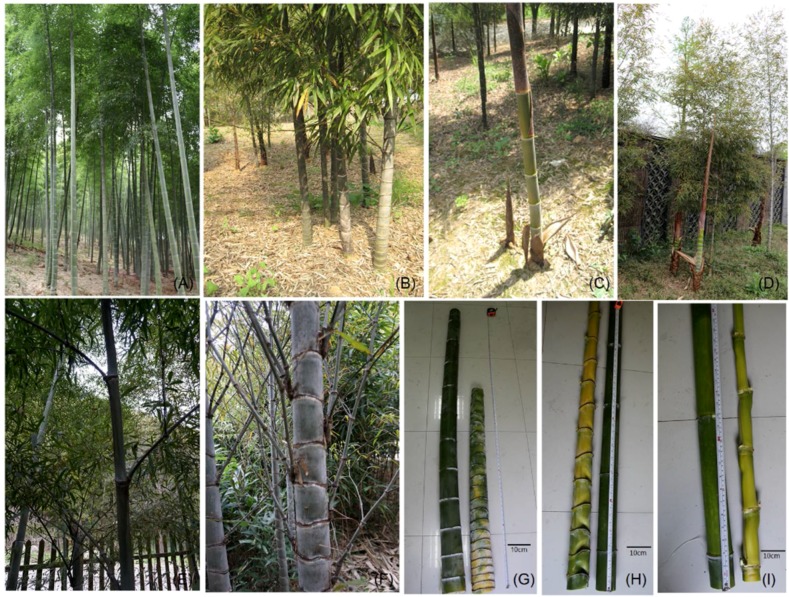
Phenotypic traits of *P. edulis* f. *tubaeformis* (dwarf shengyin bamboo) and *P. edulis* (wild moso bamboo). Wild moso bamboo forest (**A**); dwarf shengyin bamboo forest (**B**); wild moso bamboo shoot at Yiyang (**C**); dwarf shengyin bamboo shoot at Anji (**D**). Branching in wild moso bamboo (**E**) and dwarf shengyin bamboo (**F**). Comparisons of the basal stem internodes of wild moso bamboo (left) and dwarf shengyin bamboo (right) shoots (**G**); internodes in the middle part of the stem of dwarf shengyin (left) and wild moso bamboo (right) (**H**), and internodes from the top part of the stems of 1-year-old wild moso bamboo (left) and dwarf shengyin bamboo (right) (**I**).

*P. edulis* (Carr.) H. de Lehaie f. *tubaeformis* (S.Y.Wang) Ohrnberger, commonly known as abnormal bamboo or alien gourd bamboo, is a dwarf variant of moso bamboo, (shengyin bamboo in Chinese) [1]. Shengyin differs from the normal form of moso bamboo in that it has swollen short internodes on the middle-lower part of the stems, and the swelling and shortened internodes resemble a cascade of gold ingots [1,16,17] (Figure 1). Shengyin is a highly appreciated type of ornamental bamboo in China, and should be an excellent subject for scientific research into the molecular mechanism of shortened internodes in *Phyllostachys edulis*.

To better understand the unique growth pattern of dwarf shengyin bamboo, previous studies have characterized its biological and physiological growth [18], tissue culture [16], introduction and cultivation technology [17], and response to drought and water stresses [19]. Few studies of the molecular mechanisms underlying dwarfing and internode shortening in shengyin bamboo have been reported. In this study, we performed transcriptome sequencing, hormone profiling, and anatomical observations of different states of elongation and growth of the culm (basal, middle, and top internodes) between dwarf shengyin and wild-type moso bamboo to determine the possible biological mechanisms of dwarfing in shengyin bamboo. To our knowledge, such a detailed and comprehensive investigation of bamboo has not been reported to date.

## 2. Results

### 2.1. Comparison of Phenotypic Characteristics of Dwarf Shengyin Bamboo and Wild Moso Bamboo

A morphological analysis showed that four characteristics; culm length, diameter at breast height, ground diameter, and branch angle, were significantly reduced in shengyin bamboo compared to wild moso bamboo (*t*-test, *p* < 0.01). In general, the culms of wild moso bamboo were >2-fold longer than in dwarf shengyin bamboo, with almost the same number of nodes between the two types of bamboo (Table 1). The ground diameter, diameter at breast height, and branch angle of dwarf shengyin bamboo were also significantly reduced compared to wild moso bamboo (*t*-test, *p* < 0.01; Table 1).

**Figure 2:**
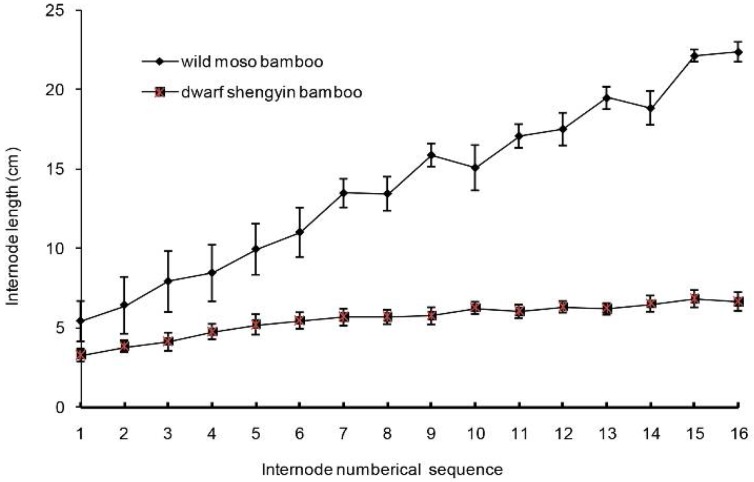
Internode lengths in the shoots of dwarf shengyin and wild moso bamboos.

To explore the internode variation in the above-ground parts of dwarf shengyin bamboo, we measured the length of each internode, starting at the bottom, from internodes 1 to 16 (Figure 2). Statistical analysis showed that each internode of dwarf shengyin bamboos was shorter than the corresponding internode in wild moso bamboo. Relative to the internode lengths in wild moso bamboo, as the internode number increased, the internode length gradually decreased in dwarf shengyin bamboo. There was no significant variation in the total number of internodes between dwarf shengyin bamboo and wild moso bamboo, but all the internodes are shorter, which results in a dwarf plant of decreased stature.

### 2.2. Vascular Anatomical Trait Observations

**Figure 3:**
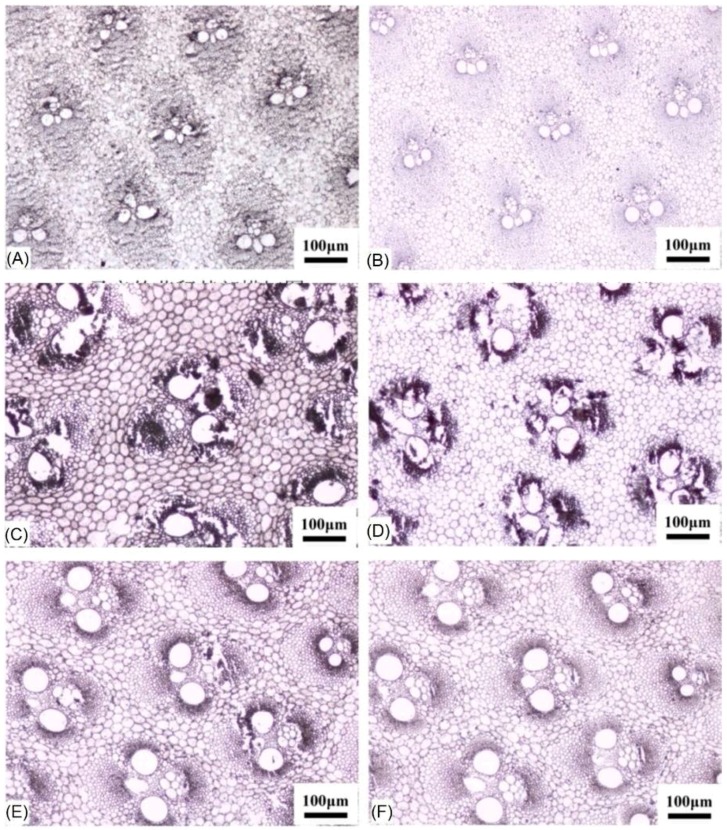
Anatomical traits of dwarf shengyin and wild moso bamboos. Transverse sections taken from the top of the culm in dwarf shengyin bamboo (**A**) and wild moso bamboo (**B**); sections from the middle of the culm in dwarf shengyin (**C**) and wild moso bamboo (**D**); and sections from the basal part of the culms in dwarf shengyin (**E**) and wild moso bamboo (**F**).

The bamboo culm consists of vascular bundles and parenchyma, and the vascular bundles are mainly responsible for determining the strength of the culm. Vascular bundles are also an important connection between the microscopic properties and the macroscopic properties of the bamboo culm. Moreover, the vascular bundle forms not only the main skeletonal structure of bamboo, but also functions in water and nutrient transport. In order to gain insight into the vascular anatomical traits of dwarf shengyin bamboo and wild moso bamboo, we measured the density, length, width, and the length-width ratio of the vascular bundles via dissection and microscopy. Examination of transverse culm sections showed that the vascular bundle densities of the two types of bamboo shoots gradually decrease from the top to the base of the culm, but that there was no significant difference between the two types of bamboo shoots in the corresponding top, middle, and basal parts (Table 2 and Figure 3). The lengths of the vascular bundles in dwarf shengyin bamboo are less than those in wild moso bamboo, and we found that the lengths at the top and middle parts of the shoots were significantly different between the dwarf and wild bamboos (Table 2). Although the width of the vascular bundles in dwarf shengyin bamboo was less than in wild moso bamboo, there was no significant difference between the dwarf and wild moso bamboos. Moreover, the length-width ratio of the vascular bundles in the basal part of the shoot in dwarf shengyin bamboo was larger than that in wild moso bamboo.

**Figure 4:**
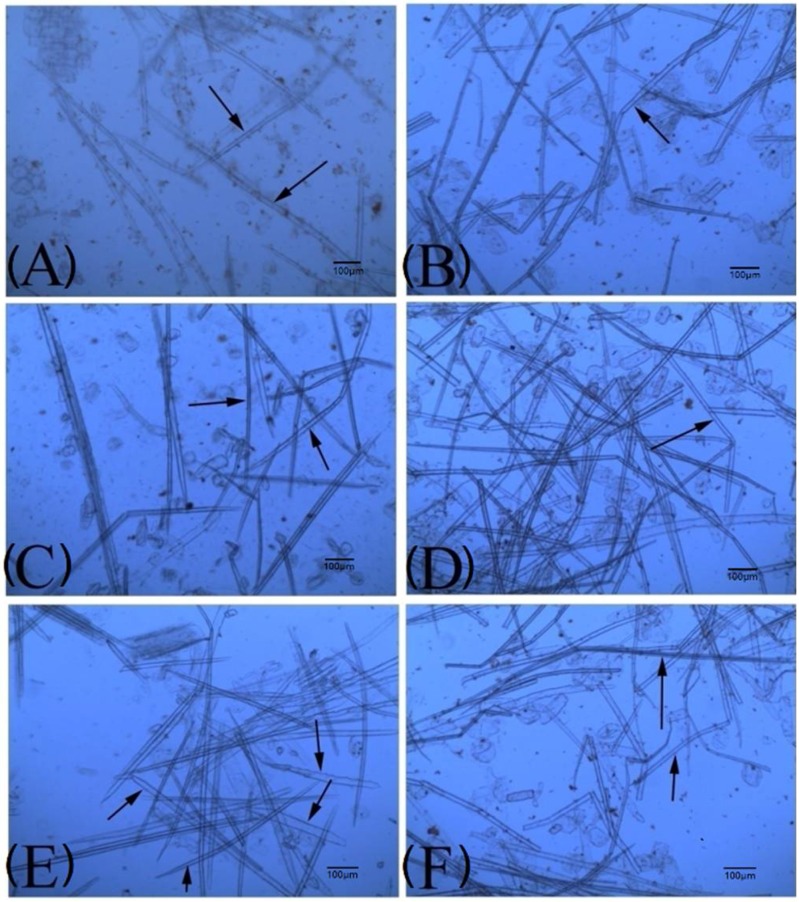
Fiber traits of dwarf shengyin and wild moso bamboos. Micrographs of fibers from the basal part of the culm in dwarf shengyin bamboo (**A**) and wild moso bamboo (**B**); the middle part of the culm in dwarf shengyin (**C**) and wild moso bamboo (**D**); and from the top of the culm in dwarf shengyin (**E**) and wild moso bamboo (**F**). The arrows indicate the fibers.

To explore the relationships between the culm variations and fiber traits in dwarf shengyin bamboo, we measured the fiber trait parameters, such as arithmetic mean length (LN), length-weighted mean length (LW), length-weighted average fiber length (LWW), width, and fines fibers percent in dwarf shengyin and wild moso bamboo, respectively. Comparison of the internode fiber traits at the same development stages (Table 3) indicated that the fiber lengths in the two bamboo plants both decreased continuously from the basal internode to the top internode. This result agreed with the previously-reported decreasing trend in fiber cell length observed from the basal internode to the top internode in developing shoots of moso bamboo [3]. Internodes of dwarf shengyin bamboo had a LWW of 0.78 mm, a very narrow fiber length distribution, and much lower fine fibers content (average approximately 28.89%), while the longer internodes of wild moso bamboo had much longer fibers (average length ~1.39 mm) and a higher fine fibers content (46.09%). The fiber length (LWW) of the same internodes between shoots of the two types of bamboo showed a significant difference, with those in dwarf shengyin bamboo being significantly shorter. Cell division and elongation occur simultaneously, affecting internode elongation [3]. Compared with wild moso bamboo, the cell numbers in the internodes of dwarf shengyin bamboo were not remarkably different, but cell size decreased in length. Also, the fiber widths in dwarf shengyin bamboo internodes were significantly higher (about 19.40 µm) than in wild moso bamboo (about 15.03 µm). These fiber trait statistics indicate that shengyin bamboo is a dwarf variant with significantly shorter and thicker fibers than wild type (Figure 4 and Table 3).

Principal component analysis was carried out by selecting traits with significant differences (*t*-test, *p* < 0.01) between dwarf shengyin and wild moso bamboos. And the results showed that most of the selecting traits might have important influence on the formation of bamboo stem traits (Figure S1).

### 2.3. Global Changes in Candidate Gene Expression during Shoot Development in Dwarf Shengyin and Wild Moso Bamboo

To gain an understanding of the possible reasons for dwarfism in shengyin moso bamboo at the transcriptional level, our goal was to identify key candidate genes that influence culm shortening. We compared transcriptome profiles of bamboo shoots at the rapid growth stage from dwarf shengyin bamboo and the native wild moso bamboo. A total of 334 million raw read pairs were generated. After removing adaptors, low quality sequences, and contaminated reads, we obtained a total of 331 million high-quality read pairs (Table 4). The cleaned reads were then mapped to the Moso genome database [20]. The transcriptome data have been deposited in the National Center for Biotechnology Information (NCBI) Sequence Read Archive under accession number SRP133731.

We identified the differentially expressed genes (DEGs) between dwarf shengyin and wild moso bamboo, and the higher number of DEGs (1345) was found in comparisons of the basal elongated internodes (SYd_vs_MZD), followed by the middle elongating internodes (220; SYb_vs_MZB). This could be due to the fact that in the mature basal elongated internodes, most of the functional cells are already relatively mature, and the larger number of DEGs represent genes that were differentially transcribed to maintain the morphologic differences between the two types of bamboo. The fewest DEGs were found in the upper parts of the plants which had not begun to elongate (72; SYa_vs_MZA) (Supplemental Table S1). The reason for this could be that the upper internodes of dwarf shengyin and wild moso bamboos are both comprised of actively growing cells, and a large fraction of the genes are likely to show similar transcriptional activity in the vigorously dividing cells. We also analyzed the DEGs between the basal elongated and the middle elongating internodes in dwarf shengyin bamboo (SYd_vs_SYb), and also in wild moso bamboo (MZD_vs_MZB), due to the generally shorter length of the basal internodes compared to the middle internodes. The results showed that 4338 DEGs were found in the MZD_vs_MZB comparison, and that is 5067 in the SYd_vs_SYb group (Supplemental Table S1).

Gene ontology (GO) enrichment analyses revealed that in the young top internodes, cell division and anatomical structure morphogenesis-related genes showed significant differential expression levels in dwarf shengyin and wild moso bamboos. In the middle stem internode regions, cellulose biosynthetic and metabolic process-related genes also showed significant differential up-regulation, and DNA/RNA synthesis-related genes were significantly down-regulated in dwarf shengyin bamboo compared with wild moso bamboo. In the mature basal part of the stem, cell wall biosynthesis- and expansin-related genes were the most significantly up-regulated DEGs in dwarf shengyin bamboo (Supplemental Table S2).

### 2.4. DEGs Involved in Cell Wall Biosynthesis and Expansion

To provide mechanical strength to newly elongated cell walls and internodes, the synthesis of new cell wall components is essential [1]. The differential expression of genes involved in cell wall synthesis or modification, such as lignin biosynthesis and cellulose synthase (CES), expansins (EXPs), xylosidase (XYL), pectate lyase (PL), pectinesterase (PM), pectin acetylesterase (PA), and polygalacturonase (PG) were assessed, because they might participate in bamboo fiber expansion.

**Figure 5:**
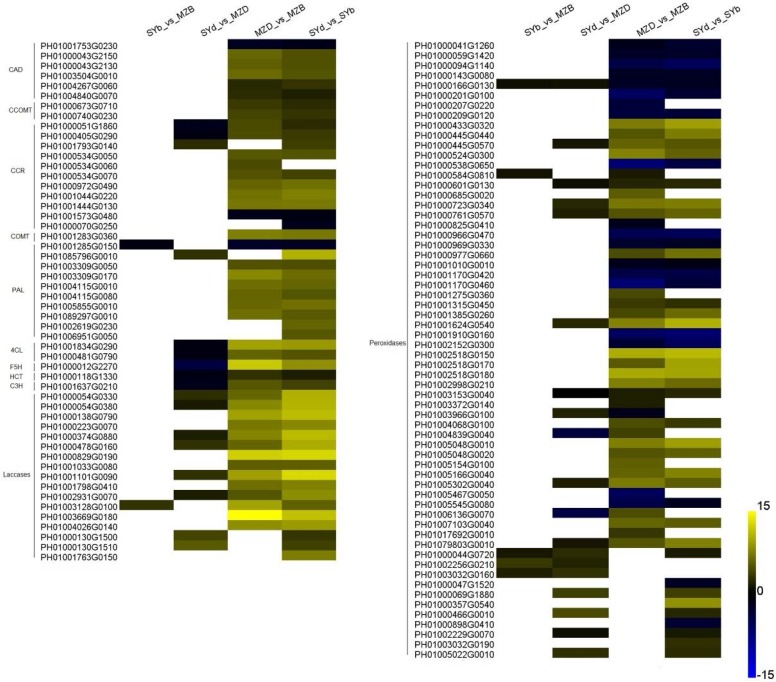
Heatmaps showing relative changes in lignin biogenesis-related gene expression between dwarf shengyin and wild moso bamboo shoots. Yellow and blue indicate up- and down-regulated transcripts, respectively, from the five comparisons, and white indicates no significant difference in expression. PAL: phenylalanine ammonia-lyase; 4CL: 4-coumarate-CoA ligase; C3H: coumarate-3-hydroxylase; F5H: ferulate 5-hydroxylase; CCR: cinnamoyl-CoA reductase; HCT: *p*-hydroxycinnamoyl-CoA: quinate/shikimate O-hydroxycinnamoyltransferase. All genes are listed in Supplementary Table S3. SYb_vs_MZB: the gene expression profiles of SYb was compared to those of MZB; SYd_vs_MZD: the gene expression profiles of SYd was compared to those of MZD; MZD_vs_MZB: the gene expression profiles of MZD was compared to those of MZB; SYd_vs_SYb: the gene expression profiles of SYd was compared to those of SYb.

Expression of the phenylalanine ammonia-lyase (*PAL*), 4-coumarate-CoA ligase (*4CL*), coumarate-3-hydroxylase (*C3H*), ferulate 5-hydroxylase (*F5H*), cinnamoyl-CoA reductase (*CCR*), and *p*-hydroxycinnamoyl-CoA:quinate/shikimate O-hydroxycinnamoyltransferase (*HCT*) genes, which are involved in biosynthesis of the three types of monolignols (*p*-coumaryl, sinapyl, and coniferyl alcohols) and are thought to play critical roles in regulating lignin accumulation in plants [21,22], were all significantly down-regulated in the basal internodes of dwarf shengyin bamboo relative to wild moso bamboo. However, expression of peroxidase and laccase genes, which participate in the oxidation and polymerization of monolignols and in the final step in the formation of lignin polymer, was significantly up-regulated in the basal and middle internodes of dwarf shengyin bamboo (Figure 5 and Supplemental Table S3).

In the basal and middle internodes of moso bamboo, most of the DEGs involved in cellulose biosynthesis were up-regulated in dwarf shengyin bamboo shoots. There are eight DGEs that encode cellulose synthase (CESA) subunits; of these, the *OsCESA3* homolog showed significantly reduced expression levels in the basal parts of dwarf bamboo, and expression levels of the other seven *OsCESA* homologous genes was significantly up-regulated in the basal or middle parts of dwarf shengyin shoots compared to wild moso bamboo. Cellulose synthase-like genes (CSLs) have been previously suggested to be involved in the biosynthesis of hemicellulose backbones [23]. In this study, the bamboo *PeCSLD2* (PH01000246G0240) gene showed significantly reduced expression levels in the shortened basal internodes of shengyin bamboo. We found that two bamboo *PeCSLC* genes were significantly up-regulated in the middle (PH01000883G0140) and basal (PH01000379G0160) internodes in dwarf shengyin bamboo at different levels relative to the expression level in wild moso bamboo (Supplemental Table S4).

**Figure 6:**
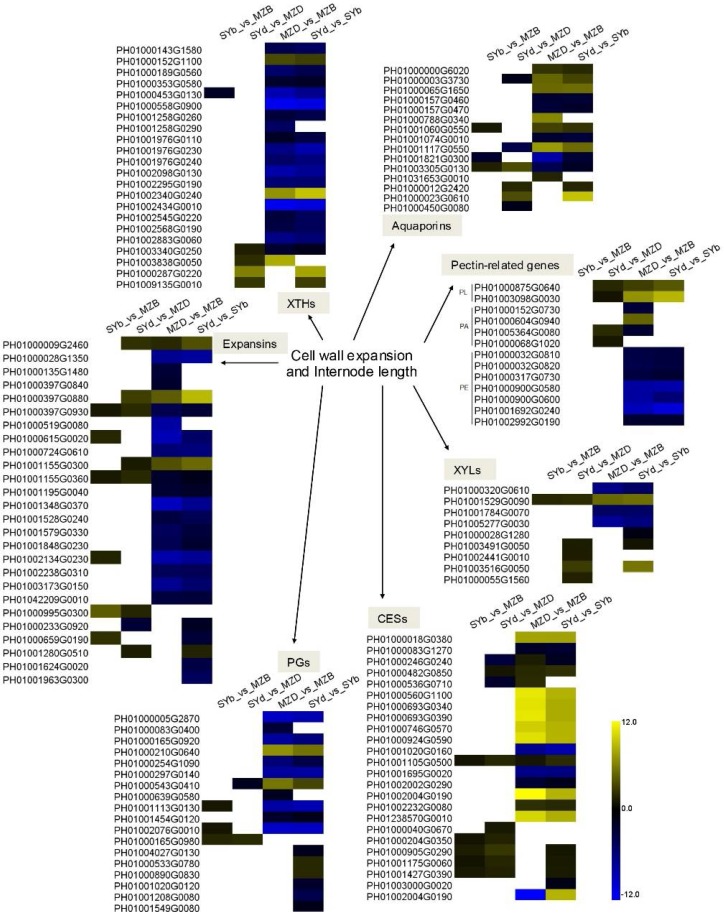
Heatmaps showing relative changes in fiber expansion-related gene expression between dwarf shengyin and wild moso bamboo shoots. Yellow and blue indicate up- and down-regulated transcripts, respectively, from the five comparisons, and white indicates no significant difference in expression. XTH: Xyloglucan endotransglucosylase/hydrolase; CES: cellulose synthase; XYL: xylosidase; PL: pectate lyase; PM: pectinesterase; PA: pectin acetylesterase; PG: polygalacturonase. All genes are listed in Supplementary Table S4. SYb_vs_MZB: the gene expression profiles of SYb was compared to those of MZB; SYd_vs_MZD: the gene expression profiles of SYd was compared to those of MZD; MZD_vs_MZB: the gene expression profiles of MZD was compared to those of MZB; SYd_vs_SYb: the gene expression profiles of SYd was compared to those of SYb.

Loosening of the plant cell wall is a necessary physiological process that occurs during cell expansion and elongation throughout the entire period of growth and development in plants [24]. The EXPs are non-hydrolytic cell wall-loosening proteins that enable cells to expand while allowing tissues to differentiate and grow [25,26]. Twenty-six putative members of the expansin gene family were differentially expressed in the bamboo internodes; 10 *EXP*s were significantly up-regulated in the shorter and thicker basal or middle internodes of dwarf shengyin bamboo (SYb_vs_MZB or SYd_vs_MZD), while 21 *EXP*s were significantly down-regulated in the shortened basal internodes of dwarf shengyin and wild bamboo (SYd_vs_SYb and MZD_vs_MZB). Xyloglucan endotransglucosylase/hydrolase (XTH) has also been shown to be involved in cell expansion by loosening and rearranging the cell wall fibers in growing tissues. Seventeen *XTH* genes were highly down-regulated in the shortened basal internodes of dwarf shengyin and wild bamboo (SYd_vs_SYb and MZD_vs_MZB); conversely, four genes were significantly up-regulated in the basal internodes of dwarf shengyin bamboo (SYd_vs_MZD; Figure 6 and Supplemental Table S4).

Two differentially expressed *PL* genes were significantly up-regulated in the basal internodes of dwarf shengyin bamboo (SYd_vs_MZD) and in the shortened basal internodes of dwarf shengyin and wild bamboo (SYd_vs_SYb and MZD_vs_MZB). Moreover, the relative expression of seven *PM*, five *PA*, nine *XYL*, and 18 *PG* genes showed significant differences.

### 2.5. DEGs Involved in Phythormone Biosynthesis and Signal Transduction Pathways

**Figure 7:**
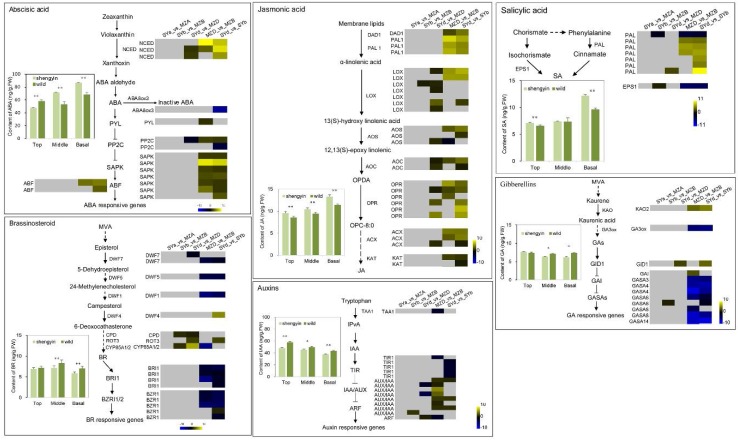
Heatmaps showing relative changes in expression of biosynthesis- and signal pathway-related genes for six phytohormones between dwarf shengyin and wild moso bamboo shoots. Yellow and blue indicate up- and down-regulated transcripts, respectively, from the five comparisons, and white indicates no significant difference in expression. The arrows indicate positive regulation, and T indicates negative regulation. SYa_vs_MZA: the gene expression profiles of SYa was compared to those of MZA; SYb_vs_MZB: the gene expression profiles of SYb was compared to those of MZB; SYd_vs_MZD: the gene expression profiles of SYd was compared to those of MZD; MZD_vs_MZB: the gene expression profiles of MZD was compared to those of MZB; SYd_vs_SYb: the gene expression profiles of SYd was compared to those of SYb. The concentrations of six endogenous hormones in the top, middle, and basal internodes of dwarf shengyin and wild moso bamboo culms are shown. Each data point represents the mean of three biological replicates and three experimental replicates. A single asterisk (*) indicates significant differences at *p* < 0.05 and two asterisks (**) indicates significant differences at *p* < 0.01 between the two genotypes according to Student’s *t*-test. Bars represent the standard error of the mean (SE; n = 9).

Recent molecular genetic studies using dwarf mutants of rice, *Arabidopsis*, and other species have revealed that some endogenous phytohormones, such as GA, BR, indole-3-acetic acid (IAA), abscisic acid (ABA), salicylic acid (SA), and jasmonic acid (JA), are also key factors in determining plant height [27]. Dwarf bamboo internode DEGs annotated as being involved in known phytohormone biosynthesis and signalling pathways are shown in Figure 7 and Supplemental Table S5.

In the GA biosynthesis pathway, expression of the *GA3ox* gene was significantly down-regulated in the shortened basal internodes of both dwarf shengyin and wild type moso bamboo (SYd_vs_SYb and MZD_vs_MZB). In the basal internodes of dwarf shengyin bamboo, the *PeGID1* (GIBBERELLIN INSENSITIVE DWARF 1, PH01001443G0490) gene showed >2-fold higher expression compared to that in wild moso bamboo. Expression of seven of the eight bamboo *GASA* homologs was reduced in the shortened basal internodes compared with the middle elongating internodes in both dwarf shengyin and wild moso bamboos (SYd_vs_SYb and MZD_vs_MZB). The exception was the expression of the GASA6 (PH01000534G0860) homolog being elevated in the shortened middle internodes of dwarf shengyin bamboo compared with wild moso bamboo (SYb_vs_MZB). In the mature basal shortened internodes of wild moso bamboo, expression of the DELLA protein GAI gene (PH01002560G0270) was >2-fold higher than it was in the middle internodes (MZD_vs_MZB). In the BR biosynthesis pathway, the genes involved in the synthesis of the upstream precursors from episterol to 6-deoxocathasterone (*DWF7*, *DWF5*, and *DWF1*) showed reduced expression in the basal shortened internodes compared with the middle elongating internodes in both dwarf shengyin and wild moso bamboos (SYd_vs_SYb and MZD_vs_MZB). The *DWF7* gene especially showed reduced expression in the basal shortened internodes of dwarf shengyin bamboo compared with wild moso bamboo (SYd_vs_MZD). The decrease in upstream precursor compounds might restrict the accumulation of downstream BR substances. However, expression of the *CPD*/*CYP90A1* (PH01003419G0030), *CYP90D2* (PH01002605G0130), and *CYP85A* (PH01001995G0390) genes was elevated in the middle and basal internodes of dwarf shengyin bamboo compared with wild moso bamboo. Expression of the BR signaling pathway genes *BRI1* and *BZR1* was also reduced in the basal shortened internodes compared with the middle elongating internodes in both dwarf shengyin and wild moso bamboos (SYd_vs_SYb and MZD_vs_MZB), and a probable BRI1 kinase inhibitor 1 gene, *BKI1*, showed a contrasting expression pattern compared with that of the *BZR1*s. In the IAA biosynthesis pathway, expression of the tryptophan aminotransferase encoding gene *TAA1*, which is involved in the indole-3-pyruvic acid (IPA) pathway of IAA biosynthesis was significantly down-regulated in the shortened basal internodes of both dwarf shengyin and wild bamboo (SYd_vs_SYb and MZD_vs_MZB). Sixteen *AUX/IAA* genes were found to have significantly different expression profiles in basal shortened internodes and middle elongating internodes. Also, three auxin efflux carrier PIN-FORMED protein-encoding genes, *PIN*s, were significantly up-regulated in the middle internodes of dwarf shengyin compared with wild moso bamboo (SYb_vs_MZB), and they were significantly down-regulated in the mature basal shortened internodes compared with the middle elongating internodes of dwarf shengyin bamboo (SYd_vs_SYb).

The expression profiles of *NCED* (9-cis-epoxycarotenoid dioxygenase) genes, which are involved in ABA biosynthesis, in bamboo basal internodes supports the significant accumulation of ABA in the mature and elongated basal internodes compared with the top and middle internodes (Figure 7 and Supplemental Table S5). Moreover, we also found that the expression of two *NCED*s (PH01001453G0450 and PH01005265G0030) was significantly elevated in the basal internodes of dwarf shengyin bamboo compared with wild moso bamboo. JA and its derivatives are lipid-derived hormones synthesized from linolenic acid [28]. Increased levels of lipoxygenase (LOX), allene oxide synthase (AOS), and allene oxide cyclase (AOC) in the plastids, OPDA reductase 7 (OPR) in peroxisomes and acyl-CoA oxidase (ACO), which are involved in JA synthesis, were detected in the basal internodes of dwarf shengyin and wild moso bamboo. Also, the expression of genes that encode enzymes that act early in JA biosynthesis were elevated in the basal internodes of dwarf shengyin bamboo compared to wild moso bamboo (Figure 7). We also examined the expression pattern of genes associated with SA biosynthesis. In bamboo, we detected significant differential expression of two *PAL* genes; PH01001285G0150 was expressed in the top and middle internodes of the two bamboo genotypes, and its expression was up-regulated in dwarf shengyin bamboo, while the expression of PH01085796G0010 was up-regulated only in the basal internodes of dwarf shengyin bamboo compared with wild moso bamboo. *EPS1* (*enhanced pseudomonas susceptibility 1*) encodes a member of the BAHD acyltransferase superfamily, and may be directly involved in the synthesis of an important precursor or regulatory molecule for SA biosynthesis [29]. In this study, the *EPS1* gene showed elevated expression in middle internodes of dwarf shengyin bamboo compared with wild moso bamboo. Some SA signaling pathway-related genes also showed significant changes in expression; expression of one *SABP* gene was elevated in the shortened basal internodes compared with the middle elongating internodes in wild moso bamboo (MZD_vs_MZB), and three *NPR*s also showed elevated expression in the shortened internodes.

### 2.6. Endogenous Hormone Concentrations

To better understand the involvement of endogenous hormones in internode length in dwarf and wild moso bamboo shoots, we quantified the concentration of six endogenous hormones, GA, BR, IAA, JA, SA, and ABA in the top, middle, and basal internodes of both genotypes using ELISA (Figure 7). The results showed that the concentrations of GA, BR, and IAA, which are often considered to be growth-promoting factors in plant development, showed declining trends from the top internode to the base of the bamboo shoots at different levels, while the levels of the hormones ABA, JA, and SA, which are usually considered to inhibit development, showed increasing trends. The changes in endogenous hormone concentrations between dwarf and wild moso bamboo were consistent with the cell division ability of the different internodes, which was in accordance with that of previous studies of different moso bamboo internodes [4]. The GA, BR, and IAA contents in dwarf shengyin bamboo were significantly lower than in wild moso bamboo on the whole. Nevertheless, the contents of ABA, JA, and SA in the internodes of dwarf shengyin bamboo were higher than in wild moso bamboo on the whole.

## 3. Discussion

### 3.1. Cell Wall Components and Internode Length

Dwarfism is often correlated with changes in cellular processes such as morphogenesis and division [30]. The fibrocytes in dwarf shengyin bamboo evidently decreased in length and increased in width compared with the same cells in wild moso bamboo. The establishment of cell size and cell shape is associated with the progress through the cell cycle, and includes crucial steps in the biogenesis of a new cell plate as well as cell-wall deposition and metabolism [30]. Moreover, some genes associated with the cytoskeleton binding proteins involved in cell division, such as tubulin, kinesin-like, MAP, and MOR1, were found to be expressed at higher levels, with different profiles, in the middle and basal internodes of dwarf shengyin and wild moso bamboo.

In general, bamboo culms contain approximately 40–60% cellulose, 16–34% lignin, and 14–25% hemicellulose with different composition ratios of the three main components at different developmental stages and in the different internodes [31]. Cellulose plays a central role in the regulation of plant cell volume and the determination of cell size [32]. The *CESA* gene family encodes the catalytic subunits of cellulose synthase and is responsible for the biosynthesis of cellulose in plant cell walls [32,33]. There are eight DGEs that encode CESA subunits, and these bamboo CESA proteins are involved in regulating cellulose biosynthesis in shengyin bamboo with distinct catalytic activities, and that more CESAs might be needed to participate in cell wall metabolism in the dwarf variant compared to wild moso bamboo. Cellulose synthase-like genes (*CSL*s) have been previously suggested to be involved in the biosynthesis of hemicellulose backbones [23]. Bamboo hemicellulose is mainly composed of xylan, and >90% [2]. Of all the *CSL* gene families, the *CSLD* genes are the most closely related in sequence to the *CESA* genes that are required for β-1,4-glucan synthesis and cell extension and elongation [23]. In this study, the bamboo *PeCSLD2* (PH01000246G0240) gene showed significantly reduced expression levels in the shortened basal internodes of shengyin bamboo. Moreover, CSLC was demonstrated to control the biosynthesis of the β-d-glucan xyloglucan backbone in nasturtium (*Tropaeolum majus*) [34]. And two bamboo *PeCSLC* genes have significantly elevated expression levels in the middle (PH01000883G0140) and basal (PH01000379G0160) internodes in dwarf shengyin bamboo with different levels relative to wild moso bamboo (Supplemental Tables S1 and S4). Lignin polymers are deposited mainly in the secondary walls of tracheary elements and fibers, and serve to strengthen plant cells and to protect the cell walls from microbial degradation. The expression of peroxidase and laccase genes, which participate in the oxidation and polymerization of monolignols, and in the final step in the formation of lignin polymer, were significantly up-regulated in the basal and middle internodes of dwarf shengyin bamboo. However, expression levels of the genes that are involved in catalyzing the formation of the three types of monolignols were all significantly down-regulated in the basal internodes of dwarf shengyin bamboo relative to wild moso bamboo. This indicates that the accumulation of monolignols in dwarf shengyin bamboo might be lower than in wild moso bamboo, and it is possible that dwarf shengyin bamboo does not produce enough lignins to support cell elongation and growth.

The cell wall determines the shape and direction of growth of individual cells as well as of whole tissues through the mechanical control of cell expansion and elongation [35]; therefore, plant growth and development requires modulation of cell size and shape, which is accomplished by regulated changes in cell wall plasticity. Plant cell wall loosening is a necessary physiological process during cell expansion and elongation throughout the entire period of growth and development in plants [24]. Expansins are non-hydrolytic cell wall-loosening proteins [25,26]. The role of expansins has been demonstrated in many plants; for example, the overexpression *AtEXLA2* decreases cell wall strength in Arabidopsis hypocotyls, *OsEXP4* significantly increases the length of rice stems, and *GmEXPB2* promotes root elongation. In addition, the poplar *PtEXPA* gene might participate in formation of the xylem. In the short-fiber cotton mutant *Ligon Lintless-1* (*Li-1*), the expression levels of several expansin genes was dramatically down-regulated. In our study, the *OsEXLA3*-homologous gene PH01000233G0920 had significantly lower expression levels in dwarf shengyin bamboo basal internodes than in wild moso bamboo, indicating that the bamboo *EXLA3* gene may participate in increasing internode length by enabling cell expansion. Although it has long been believed that expansins play positive roles in plant height and tissue enlargement, this is in fact not the case. In leaves of *Festuca pratensis* (meadow fescue), there was no correlation between the expression level of *EXP*s and the incremental increase in blade length. The overexpression of expansin genes in transgenic rice and tomato resulted in dwarfism, and transgenic tobacco plants overexpressing expansin genes from Chinese fir showed drastic increases in stem diameter [36]. In hybrid aspen (*Populus tremula* L. × *P. tremuloides* Michx.), *PtEXPA1* expression stimulated fiber diameter growth [26]. These results are consistent with the observed elevated expression levels of ten *EXP* genes in the middle and basal internodes of dwarf shengyin bamboo compared with wild moso bamboo. And the fiber diameter in the dwarf shengyin bamboo internodes was significantly thicker (about 19.40 µm) than that of wild moso bamboo (about 15.03 µm). These results show that increasing the expression level of expansin genes may not always increase plant height, and could possibly cause transverse cell expansion to increase fiber or stem diameter in plants.

*XTH* genes are another group of candidates for endogenous cell wall-loosening factors because they function to mediate cleavage and rejoining of the β (1-4)-xyloglucan cross-links between cellulose microfibrils of the primary growing cell wall [25]. Growth-promoting phytohormones have also been found to induce *XTH* gene expression; for example, the *AtXHT* and soybean *BRU1* genes are highly and rapidly induced by BR-treatment during the early stages of elongation, *OsXHT* is up-regulated by GA to be involved in cell elongation, and auxin regulates *XHT* expression in expanding tomato hypocotyls [25]. In this study, the *AtXTH28*-homologous gene PH01000453G0130 showed high level tissue-specific expression in the top internodes where cells actively proliferate with almost equal expression levels in the dwarf and wild moso bamboos, indicating that it is involved in cell rapid differentiation and expansion in the shoot apical meristem in bamboo. Moreover, it has significantly lower expression levels in the middle internodes of dwarf shengyin bamboo than in wild moso bamboo, and no expression in basal internodes from both bamboo types, indicating that XTH promotes the early stages of internode elongation in bamboo. While there are four probable *XTH* genes (Supplemental Table S4) that show high-level expression, they also show significantly elevated expression levels with different profiles in dwarf shengyin bamboo internodes compared with wild moso bamboo. These genes also participate in transverse cell expansion to increase the fiber or stem diameters in dwarf shengyin bamboo.

### 3.2. The Role of Plant Hormones in Determining Internode Length

Plants that are deficient in the biosynthesis of growth-promoting hormones, or that are defective in signal transduction, show abnormal dwarfed developmental phenotypes, indicating the importance of both endogenous phytohormone biosynthesis and the signaling pathways in regulating the processes of cell and stem elongation, vasculature differentiation, and cellulose biosynthesis [27].

In rice and other species in the grass family, GA-deficient or GA-insensitive mutants show dwarf phenotypes [10,37]. The GID1 protein has been hypothesized to be a GA soluble positive receptor molecule in the GA signaling pathway. In the basal internodes of dwarf shengyin bamboo, the *PeGID1* gene showed higher expression levels compared to that in wild moso bamboo, which is contrary to the fact that loss of function mutations in this gene result in plants that display dwarf phenotypes and GA insensitivity [37]. However, our finding is in agreement with the GA-triggered negative feedback loop that represses transcription of *GID1* genes in *Lepidium sativum* [35], and is supported by the reduced expression levels of *GhGID1-1* and *GhGID1-2* by GA in cotton [38]. Expression levels of the *GASA6* homolog in moso bamboo were elevated in basal elongated internodes of wild moso bamboo compared with the shortened internodes in dwarf shengyin bamboo, and our results support the idea that GASAs (GA-stimulated transcripts in *Arabidopsis*) act as positive regulators for GA-dependent processes, which is also demonstrated by the positive effects of GASA6 on floral development, GASA4 on seed germination, and GASA14 on vegetative growth [39]. The results of two previous studies suggest that GASA6 could be involved in complex hormone and glucose signaling crosstalk [39,40]; we found that the expression profile of *GASA6* in the middle internodes was opposite to that in the basal internodes, and this might be because GASA6 is differentially regulated by many other growth- and stress-related hormones.

In Arabidopsis and rice, for example, a number of BR-deficient mutants mapping to different loci have been identified as BR-related mutants by their distinctive dwarf phenotypes. Examples of BR synthesis-deficient-related mutants are *D2* (a rice dwarf mutant, *dwarf2*) [41], *cpd* [42], and *brd1* (BR-deficient dwarf 1) [43]. The *OsD2* gene encodes CYP90D2/ROT3, which catalyzes the steps of BR C-3 oxidation (i.e., reactions converting 6-deoxoTE to 6-deoxo3DT and TE to 3DT) [43]. The *AtCPD* gene encodes C-23-hydroxylase CPD\CYP90A1, which was also found to be negatively regulated by BRs at the transcriptional level [41]. The *Arabidopsis cpd* mutant displays reduced epidermal cell file length and plant dwarfism [42]. The BR-deficient dwarf *brd1* mutation is caused by the loss of function of a gene that encodes a protein homologous with the OsDWARF and *Arabidopsis* CYP85A proteins, which catalyze the C-6 oxidation step in BR biosynthesis [9]. Elevated *OsDWARF* gene expression was detected in the *brd1* dwarf mutants compared to the wild-type controls, indicating that the expression of *OsDWARF* is negatively regulated by the BR signal transduction pathway. The bamboo *CPD*/*CYP90A1*, CYP90D2/ROT3, and CYP85A genes also showed elevated expression levels in the middle and basal internodes of dwarf shengyin bamboo compared with wild moso bamboo, which was unexpected, given the lower level of BR in dwarf shengyin bamboo compared with wild moso bamboo. This indicates that the expression levels of *ROT3/CYP90D2*, *CPD*/*CYP90A1*, and *CYP85A* could be negatively regulated by the BR signal transduction pathway in dwarf shengyin bamboo, corresponding with the homologous genes in rice and *Arabidopsis*. The second factor causing dwarfing in BR-related mutants is a defect in signaling components. Defects in BR recognition and signal transduction, and the abnormal activation or deactivation of signal components can disrupt BR responses and homeostasis [9]. The GSK2/SHAGGY-like kinase is likely to be the rice counterpart of Arabidopsis BIN2, and overexpression of GSK2 led to plants with typical BR loss-of-function phenotypes. One of the bamboo GSK2-like genes (PH01003893G0130) showed elevated expression levels in the basal internodes of dwarf shengyin and wild moso bamboo compared with that in the elongating middle internodes, which did not agree with the lower level of BR, implying that bamboo *GSK2* might be involved in the regulation of dwarfing with a negative role in BR signaling. The expression of *GSK2* is negatively regulated by the BR signal and inhibits transcription of *BZR1*, along with GA biosynthesis and signal pathways to co-regulate cell elongation and plant height in rice.

Expression of the ABA biosynthesis gene *NCED*, which codes for the enzyme catalyzing oxidative cleavage of cis-epoxycarotenoids [44], was detected. The expression profiles of *NCED* genes in bamboo basal internodes supports the significant accumulation of ABA in the mature and elongated basal internodes compared with the top and middle internodes (Figure 7 and Supplemental Table S5). Moreover, two *NCED* genes showed significantly elevated expression levels in the basal internodes of dwarf shengyin bamboo compared with wild moso bamboo; this is in agreement with the trend of ABA content found in the two bamboo genotypes. It seems that there is a positive correlation between the accumulation of endogenous ABA and the shortened basal internodes in dwarf shengyin bamboo.

There are another two endogenous inhibitory hormones, JA and SA. Athough JA and SA signaling is related to pathogen defense and responses to abiotic stress, many studies have suggested that these hormones also participate in plant development [45,46]. JA has been reported to favor the accumulation of intracellular sucrose, and to increase the thickness of microtubules and microfilaments [46]. With increasing JA concentrations, the main roots of potato (*Solanum tuberosum*) plantlets became shorter and thicker [45]. Continuous exogenous JA application inhibited cotton fiber elongation in vitro and in vivo, indicating the negative relationship between JA and fiber elongation [47], and that JA could be a significant regulator in the control of elongation growth in plants [11,48]. Compared to wild moso bamboo, the relative levels of the growth promoting hormones GA, IAA, and BR were reduced in dwarf shengyin bamboo, while levels of inhibitory hormones, such as JA, were increased. This result was also supported by the finding that JA probably inhibits stem elongation in *Pharbitis nil* [49]. Exogenous JA application not only reduced the effect of GA on the elongation of both the second leaf sheath of dwarf rice seedlings and lettuce hypocotyls [50], but also reduced IAA-induced elongation of etiolated oat coleoptiles by interfering with some aspects of sugar metabolism that are related to the degradation and/or the synthesis of cell wall polysaccharides [11].

SA is another important signal molecule in plants. Young tissues (apex buds and young leaves) of dwarf cultivars of hop (*Humulus lupulus*) plants have been found to have considerably higher levels of SA compared to normal cultivars [13]. The differences in SA contents between normal and dwarf cultivars could be related mainly to the dwarf growth habit, which is consistent with recently published data showing high levels of SA in Arabidopsis dwarf mutants and growth inhibition of chamomile plants by exogenous SA application [51]. More direct evidence supporting the key role of endogenous SA in the regulation of plant cell growth comes from the characterization of Arabidopsis mutants or transgenic plants affected in the SA signaling pathway [52,53], and SA is a negative regulator of cell division through interaction with multiple receptors or signaling pathways that control cell growth and development [54]. SA binding proteins, such as SABPs and NPR1, play key roles in transmitting the SA signal in plants. NPR1, as the key factor in SA signal transduction, down-regulates the SA levels [51]. Expression levels of the *SABP* and *NPR1* genes were elevated in dwarf shengyin bamboo internodes compared to wild moso bamboo.

The synergy and antagonism between phytohormones play essential roles in controlling plant height. Previous studies have shown that GA, BR, and IAA can promote cell elongation in internode tissue [37,41,42], and act as regulators of stem elongation [43,54]. In addition, exogenous JA inhibits cotton fiber elongation [47], and high levels of SA were detected in Arabidopsis dwarf mutants [51]. Many dwarf mutants have been characterized as being either deficient in or insensitive to endogenous hormones due to mutations in biosynthesis- or signaling pathway-related genes [27].

**Figure 8:**
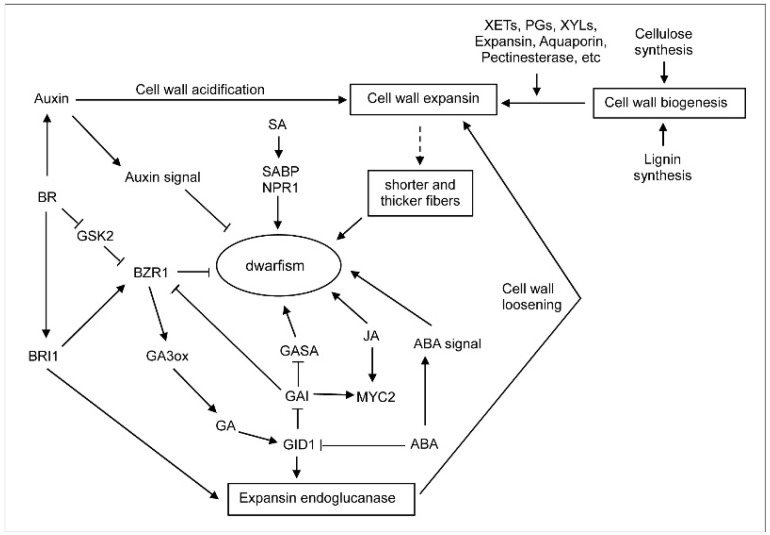
A proposed model showing the complex cross-talk between phytohormone signaling cues and cell wall expansion that could lead to dwarfism in shengyin bamboo.

The basic GA signal transduction pathway, the GA-GID1-GAI pathway, has been identified in rice and many other plants [55,56,57], and may be similar to that in bamboo. We have also found that the expression levels of some *GASA* genes, which act downstream of DELLA [58], were reduced in the shortened internodes. This agreed with previous results in *Arabidopsis*, in which the homologous *GASAs* showed down-regulated expression levels, with *GAI* expression levels up-regulated, in the growth-inhibited plants [58]. We therefore speculate that there might be a GA-GID1-GAI-GASA pathway involved in the regulation of dwarfing in shengyin bamboo (Figure 8). In the BR signal transduction pathway, BR could negatively influence the dwarfing in shengyin bamboo by directly or indirectly facilitating the expression of *BRI1* and *BZR1*, and inhibiting the expression of the *GSK2* gene. The BR and GA pathways are integrated through crosstalk between BZR1 and the DELLA protein GAI, which represses the regulatory activities of BZR1 by inhibiting BZR1 binding to the promoters of target genes [55,56]. BR also can enhance GA abundance by increasing the expression of the GA biosynthetic gene GA3ox to promote growth; thus, GA homeostasis is maintained via feedback regulation of GA levels through major target genes like *GA3ox* in rice [55]. ABA could act through a signaling pathway to promote dwarfing in shengyin bamboo, and may also inhibit the expression of GID1, which is involved in the GA signaling pathway, to promote dwarfing in shengyin bamboo. The crosstalk between GA and JA confers pathogen resistance by modulating plant growth, and GA and JA might also act antagonistically to modulate dwarfing in bamboo. The accumulation of the DELLA protein GAI in turn inhibits GA-promoted shoot elongation, which is beneficial to the release of the transcription factor MYC2 and enhancement of the JA-mediated pathway [55,56].

Based on previous studies and our analysis of the data, we have also hypothesized that complex regulation of various phytohormone signaling pathways that are possibly involved in coordination of cell wall biosynthesis and expansion could eventually lead to overall shortened fibers and reduced plant height [59]. IAA might facilitate cell wall expansion by mediating cell wall acidification, and expansins, XETs, PGs, XYLs, PLs, PEs, PAs, and aquaporin can also facilitate cell wall expansion (Figure 8). The BR signaling pathway factor BRI1 and the GA receptor GID1 could regulate the activity of some endogenous cell wall-loosening factors (e.g., endoglucanases), which function to mediate cleavage and rejoining of the beta (1-4)-xyloglucan cross-links between cellulose microfibrils in the primary growing cell wall [25].

## 4. Conclusions

Here, the biological mechanisms that determine shortened culms in a dwarf variant of moso bamboo (shengyin bamboo) have been studied for the first time. The phenotypic and anatomical observations revealed that the fibers have reduced length and increased diameter in dwarf shengyin bamboo. Comparative transcriptomic analyses provided insights into the molecular mechanisms behind shorter internodes and the reduced length and increased diameter of the fibers, particularly those processes involved in cell wall loosening, biosynthesis of lignin and cellulose, and endogenous hormone biosynthesis and signal transduction. However, reduced internode length and the associated dwarf phenotype is a complicated developmental process involving numerous factors. In the near future, more extensive studies with well-defined gene structural variants and functional verification will be necessary to validate some of the conclusions drawn from our research.

## 5. Materials and Methods

### 5.1. Plant Materials

Young shoots with 36–39 internodes approximately (2.57 ± 0.329) m above ground height of wild moso bamboo (*P. edulis*) and (1.62 ± 0.017) m above ground height of dwarf shengyin bamboo (*P. edulis* f. *tubaeformis* S.Y.Wang) emerging from the ground were harvested in May 2014 at the breeding base of Shengyin bamboo resources in the Yiyang Research Institute of Forestry Science of Hunan Province, China (coordinates 28°30′ N, 112°30′ E). The bamboos used in this study were in the boosting stage [7]; at this stage, dwarf shengyin and wild moso bamboo shoots have completely elongated basal internodes, elongating middle internodes, and top internodes that have not initiated elongation. In total, 30 shoots of approximately the same size and developmental stage were collected. Each bamboo shoot was dissected to sample the basal, middle, and top parts by height, with the basal 1–2 internodes above the ground surface, the middle 11–12 internodes, and the top 36–37 internodes, respectively. The same shoots were used for transcriptome sequencing, and hormone analyses.

### 5.2. Phenotypic Investigations

We selected 30 dwarf shengyin bamboo and wild moso bamboo plants at random from the breeding base, and measured individual plant heights, the length of each node, ground diameter, diameter at breast height, number of nodes of the main stem, nodes under branch, and branch angles.

### 5.3. Anatomical Characteristics

For young bamboo shoots, the top, middle, and basal internodes were cut into 0.5 cm^3^ sections and divided into two groups; one group was fixed in buffered formalin acetic-70% alcohol (FAA, *v*/*v*) and exhausted with an aspirator pump. Paraffin sections were made as described previously [60]. Subsequently, these sections were observed under a CX21-MDS300 (Olympus, Tokyo, Japan) light microscope. Ten circular areas, 5 mm in diameter in cross section (three duplicates per shoot), were picked randomly to analyze the density distribution of the vascular bundles. The other group of shoot samples was used to investigate the properties of single bamboo fibers isolated by the nitric acid and potassium chlorate method [61]. Fiber characteristics were observed under a Fiber Quality Analyzer (Code LDA02). We measured 5000 fibers each time. The length-related terms are defined as follows: arithmetic mean length (LN = ∑n_i_L_i_/∑n_i_); length weighted mean length (LW = ∑n_i_L_i_^2^/∑n_i_L_i_); weight weighted mean length (LWW = ∑n_i_L_i_^3^/∑n_i_L_i_^2^) [62].

### 5.4. cDNA Library Construction and Transcriptome Sequencing

Transcriptome sequencing was performed on the Illumina HiSeq 2000 system. Total RNA from the samples (each group consisted of mixed internodes from five shoots) described above was isolated using TRIzol Reagent (Invitrogen, Carlsbad, CA, USA). cDNA libraries were constructed, and 125 bp/150 bp paired-end reads were generated on the Illumina Hiseq 2000 instrument. All raw reads were first processed to remove the adaptor sequences, low-quality reads, and possible contamination from chloroplasts, mitochondria, and ribosomal DNA. The clean reads were then aligned to the moso bamboo genome sequence using TopHat v2.0.12 (University of Maryland, College Park, MD, USA) [63] to identify exons and splice junctions. HTSeq v0.6.1 (University of California, Berkeley, San Francisco Bay Area, CA, USA) was used to count the numbers of reads that mapped to each gene, which were normalized to fragments per kilobase of exon per million fragments mapped (FPKM) [64]. The read counts for each library were adjusted using the edgeR program package for differential gene expression analysis. The DEGSeq R package (1.20.0; Jilin University, Changchun, China) was used for differential expression analysis of two conditions [65]. The *p*-values were adjusted using the Benjamini & Hochberg method. Corrected *p*-values of 0.005 and log2 (Fold-change) of 1 were set as the threshold for significantly differential expression. GO enrichment analysis of DEGs was implemented in the GOseq R package, which was corrected for gene length bias [66]. GO terms with corrected *p*-values < 0.05 were considered significantly enriched in the DEGs.

### 5.5. Quantification of Endogenous Hormones in Bamboo Shoots

The endogenous phytohormones IAA, GA, ABA, BR, SA, and JA in the top, middle, and basal parts of the shoots were extracted and purified as described previously [67]. Enzyme-linked immuno sorbent assays (ELISA) were used to estimate the hormone levels with three biological replicates for each set of experiments.

## Figures and Tables

**Table 1:** Comparison of phenotypic traits between dwarf shengyin and wild moso bamboos.

Phenotypic Traits	Dwarf Shegnyin	Wild	*p*-Value
Plant height (cm)	415.23 ± 28.63	914.21 ± 45.99	0.000 **
Ground diameter (cm)	4.23 ± 0.29	5.03 ± 0.3	0.000 **
Diameter at breast height (cm)	3.32 ± 0.11	4.18 ± 0.16	0.000 **
Nodes of main stem	35.37 ± 1.49	36.47 ± 2.17	0.056
Nodes under branch	19.11 ± 2.69	20.37 ± 2.78	0.083
Branch angle (°)	31.56 ± 4.64	41.39 ± 4.31	0.000 **

± indicates the SD, ** *p*-value < 0.01.

**Table 2:** Comparisons of vascular bundle parameters between dwarf shengyin and wild moso bamboos.

Index	Position	Dwarf Shengyin	Wild	*p*-Value
Vascular bundle density	Top part	411.41 ± 18.06	405.78 ± 21.41	0.118
	Middle part	373.59 ± 11.59	367.34 ± 13.68	0.051
	Basal part	227.97 ± 10.84	231.56 ± 15.99	0.089
Vascular bundle length (μm)	Top part	245.26 ± 9.88	257.17 ± 29.26	0.0345 *
	Middle part	293.59 ± 20.3	314.09 ± 22.54	0.0451 *
	Basal part	430.39 ± 42.25	436.57 ± 12.68	0.689
Vascular bundle width (μm)	Top part	176.08 ± 19.35	186.09 ± 21.32	0.694
	Middle part	200.34 ± 20.19	207.67 ± 17.73	0.782
	Basal part	234.76 ± 19.07	243.15 ± 20.2	0.764
Length-width ratio of vascular bundle	Top part	1.41 ± 0.15	1.39 ± 0.16	0.581
	Middle part	1.48 ± 0.16	1.53 ± 0.19	0.671
	Basal part	1.84 ± 0.16	1.81 ± 0.15	0.801

± indicates the SD, * *p*-value < 0.05, *N* = 90 (N is sample number).

**Table 3:** Culm internode fiber characteristics of dwarf shengyin and wild moso bamboos.

	Dwarf Shengyin				Wild				*p*-Value
Internode	Top	Middle	Basal	Average	Top	Middle	Basal	Average	
LN (mm)	0.336	0.343	0.331	0.337	0.335	0.443	0.294	0.357	0.282
LW (mm)	0.526	0.542	0.509	0.527	0.723	0.924	0.607	0.751	0.000 **
LWW (mm)	0.796	0.803	0.738	0.780	1.357	1.639	1.169	1.388	0.000 **
Fine Fiber Percent (%)	29.396	28.567	28.702	28.888	47.004	41.638	49.638	46.093	0.000 **
Width (µm)	17.911	20.956	19.344	19.404	14.944	14.878	15.256	15.026	0.000 **

LN: arithmetic mean length, LW: length-weighted mean length, LWW: length-weighted average fiber length. ± indicates the SD, ** *p*-value < 0.01. Significant differences in parameters among the parts were analyzed by ANOVA and Independent-Sample *t*-Test.

**Table 4:** Summary of Illumina transcriptome sequencing data for wild moso and dwarf shengyin bamboos.

Sample	Raw Reads (bp)	Clean Reads (bp)	Clean Bases	Error Rate (%)	Q20 (%)	Q30 (%)	GC Content (%)
Dwarf shengyin_top (SYa)	43,415,772	42,548,904	4.26 Gb	0	100	100	51.68
Dwarf shengyin_middle (SYb)	45,744,478	45,148,166	4.51 Gb	0	100	100	51.51
Dwarf shengyin_basal (SYd)	41,475,324	40,879,456	4.09 Gb	0	100	100	53.43
Wild_top (MZA)	42,958,664	42,324,442	4.24 Gb	0	100	100	51.89
Wild_middle (MZB)	40,210,766	40,116,284	4.01 Gb	0	100	100	50.97
Wild_basal (MZD)	37,975,966	37,871,040	3.79 Gb	0	100	100	54.53

## Supplementary Materials

The following are available online at http://www.mdpi.com/1422-0067/19/6/1697/s1.

## Abbreviations

4CL	4-coumarate-CoA ligase
ABA	Abscisic acid
ACO	Acyl-CoA oxidase
AOC	Allene oxide cyclase
AOS	Allene oxide synthase
BR	Brassinosteroid
*brd1*	BR-deficient dwarf 1
BRI1	Brassinosteroid Insensitive 1
BZR1	BRASSINAZOLE-RESISTANT 1
C3H	Coumarate-3-hydroxylase
CCR	Cinnamoyl-CoA reductase
CES	Cellulose synthase
CSL	Cellulose synthase-like genes
*D2*	A rice dwarf mutant, dwarf2
DEG	Differentially expressed genes
DELLA	D-aspartic acid, E-glutamic acid, L-leucine, A-alanin
*EPS1*	Enhanced pseudomonas susceptibility 1
EXP	Expansin
F5H	Ferulate 5-hydroxylase
GA	Gibberellin acid
GAI	Gibberellin acid insensitive
GASA	GA-Stimulated in Arabidopsis
GO	Gene ontology
GID1	GIBBERELLIN INSENSITIVE DWARF 1
HCT	p-hydroxycinnamoyl-CoA:quinate/shikimate O-hydroxycinnamoyltransferase
IAA	Indole-3-acetic acid
IPA	Indole-3-pyruvic acid
JA	Jasmonic acid
LN	Arithmetic mean length
LOX	Lipoxygenase
LW	Length-weighted mean length
LWW	Length-weighted average fiber length
MYC2	Myelocytomatosis protein 2
MZA	Top internodes of wild moso bamboo
MZB	Middle internodes of wild moso bamboo
MZD	Basal internodes of wild moso bamboo
NCED	9-*cis*-epoxycarotenoid dioxygenase
NPR1	Nonexpressor of pathogenesis-related genes 1
OPR	OPDA reductase 7
PA	Pectin acetylesterase
PAL	Phenylalanine ammonia-lyase
PG	Polygalacturonase
PL	Pectate lyase
PM	Pectinesterase
SA	Salicylic acid
SABP	SA binding protein
SYa	Top internodes of dwarf shengyin moso bamboo
SYb	Middle internodes of dwarf shengyin moso bamboo
SYd	Basal internodes of dwarf shengyin moso bamboo
XYL	Xylosidase
XTH	XYLOGLUCAN endotransglucosylase/hydrolase
